# Effect of Pulsing Digital Heating Devices on Skin Parameters, Subjective Pain, Mood, and Anxiety

**DOI:** 10.3390/jcm12237206

**Published:** 2023-11-21

**Authors:** Nicole Natarelli, Chaitra Subramanyam, Nimrit Gahoonia, Waqas Burney, Raja K. Sivamani, Jessica Maloh

**Affiliations:** 1 Morsani College of Medicine, University of South Florida, Tampa, FL 33620, USA; natarellin@usf.edu; 2 College of Osteopathic Medicine, Western University of Health Sciences, Lebanon, OR 97355, USA; chaitra@integrativeskinresearch.com; 3 Integrative Skin Science and Research, Sacramento, CA 95815, USA; ngahooni@student.touro.edu (N.G.); waqas@integrativeskinresearch.com (W.B.);; 4 College of Osteopathic Medicine, Touro University, Vallejo, CA 94592, USA; 5 Pacific Skin Institute, Sacramento, CA 95815, USA; 6 College of Medicine, California Northstate University, Elk Grove, CA 95757, USA; 7 Department of Dermatology, University of California-Davis, Sacramento, CA 95616, USA

**Keywords:** heat, pain, mood, anxiety, TEWL, heating device

## Abstract

A common pitfall of many conventional heat therapy methods is the propensity to lose heat over time and the need for reheating and reapplication. Pain-relieving digital heating devices are now available that can be held in place on the body via adhesive or magnet and provide pulsed heat. However, the safety of such devices among different ages and skin types must be established. We conducted a prospective, open-label study to assess the effect of three consecutive thirty-minute treatment cycles on skin parameters and pain. Effects on mood and anxiety were secondarily assessed. 22 adult participants (20 female, 2 male; mean 58 ± 17.63 years) were recruited. The participants attended one visit with heating device intervention and a follow-up visit after 7–10 days. A 97% significant increase in transepidermal water loss was observed immediately following intervention (*p* = 8.04487 × 10^−7^), although significance was not sustained at follow-up. There was an increase along the red/green axis at 13/14 treatment locations immediately following treatment, although only four locations remained significantly increased at follow-up. Pain non-significantly decreased immediately following treatment (*p* = 0.057). A 38.6% decrease in subjective anxiety was observed immediately after treatment (*p* = 0.0139), and scores remained non-significantly reduced at follow-up (19.3% reduction from baseline; *p* = 0.1397). The heating devices elicit temporary changes to the skin, although the lack of significance at follow-up suggests that the devices can be safely used without long-term changes in skin color or barrier status.

## 1. Introduction

Heating pads and devices have conventionally been used for pain reduction. Heat therapy provides an alternative intervention for pain relief, reducing reliance on conventional treatments such as opioids, acetaminophen, and nonsteroidal anti-inflammatory medications (NSAIDs), which may have limited efficacy, side effects, or complication risk [1]. Physiological effects and pain reduction are mediated by increased blood flow, metabolism, and elasticity of connective tissues, with evidence from randomized controlled trials suggesting heat-wrap therapy provides significantly greater pain relief for delayed-onset muscle soreness than cold therapy [2]. Furthermore, a 2021 systematic review found heat treatment could reduce pain in patients within 24 h, with a standardized mean difference of −1.17 (95% confidence interval −2.62 to −0.09, *p* = 0.03), and over 24 h, with a standardized mean difference of −2.31 (95% confidence interval −2.97 to −0.59, *p* = 0.003 [3]). Specific thermal therapies, such as hot pack application, hot water immersion, ultrasound, and sauna, demonstrated varying efficacy, with hot pack application being the most efficacious. However, one common pitfall of topical heat therapy methods, including warm compresses, hot stone massages, and capsaicin-containing ointments, is the propensity to lose heat over time and the need for reheating and reapplication. Digital heating devices are now available and can be held in place on the body through an adhesive or magnet to provide pulsed heat. Their intended use is for the management of pain, and the small, circular devices allow for localized heating.

These user-friendly digital heating devices (The Soovu System, Soovu Labs, Inc., Seattle, WA, USA) allow for control of heat delivery via a mobile application, localized application, and pulsatile delivery of heat, which has been demonstrated to provide more effective pain relief than steady heat for low back pain [4]. Prior studies have demonstrated that these heating devices effectively deliver fast pain relief [5], lasting pain relief [4], and allow for in-home use while simultaneously engaging in activities of daily living [6].

Resulting analgesia is hypothesized to occur via activation and sensitization of TRPV1 receptors, also known as capsaicin receptors [1]. TRPV1 receptors are located throughout the body on afferent sensory fibers, C and A-delta fibers, and dorsal root ganglia. Receptor activation results in calcium and sodium influx, with resulting depolarization of nociceptive neurons and the sensation of pain [1]. However, continued activation can result in nociceptive desensitization and defunctionalization, effectively blocking action potential depolarization and pain transmission. Heat greater than 40 °C is postulated to activate TRPV1 receptors [1], justifying Soovu System heating temperature capabilities of up to 45 °C.

While pulsatile delivery of heat has been shown to be more effective than continuous heat for low back pain [4], incorporating a pulsing function may also increase patient safety and reduce total thermal energy requirements [1]. In addition, some research suggests that the use of heating devices may also be beneficial in managing mental state and anxiety [7].

Despite these potential benefits, the safety of pulsatile heating devices still needs to be established in different ages and skin types. As such, we conducted an open-label prospective study to assess the effect of three consecutive thirty-minute treatment cycles on skin parameters and pain. In addition, we assessed any shifts in mood and anxiety with the use of the heating devices, as a prior 2022 study found that heating therapy reduced both subjective and objective anxiety among those undergoing cystoscopy vs. the control group [7]. The devices are typically intended for a maximum of two consecutive treatment cycles, although three were employed for the assessment of device safety in this study. The primary objective of this study is to assess the safety of focal, pulsing heating devices applied directly to the skin by measuring transepidermal water loss (TEWL), an indicator of skin barrier integrity, and skin color for the assessment of erythema and pigmentation. The secondary objective is to assess the effect of digital heating devices on subjective pain, mood, and anxiety.

## 2. Materials and Methods

A prospective open-label study was conducted from July 2022 to October 2022. Twenty-two adult participants aged 18 years or older were recruited (20 female, 2 male; mean 58 ± 17.63 years). This study was approved by the Allendale IRB (17 June 2022). All participants provided written informed consent prior to study enrollment and were compensated for their participation. Exclusion criteria included pregnancy, peripheral neuropathy, or any neuropathic condition that could alter pain perception. Participants were not screened or excluded from the study based on the presence of a baseline psychiatric disorder. The participants attended one study visit with the heating device intervention and a follow-up visit after 7–10 days.

Prior to the placement of heating devices at the first study visit, each location underwent digital photography, TEWL measurement, and skin colorimeter measurement. TEWL was measured with a VapoMeter (Delfin Technologies Ltd., Kuopio, Finland), a non-invasive, minimal risk device that requires skin contact for approximately 15 s. Skin pigment and erythema were measured with the SkinColorCatch (Delfin Technologies Ltd., Kuopio, Finland).

In addition, subjects completed pain and mood surveys to assess baseline tendencies and changes after intervention. Subjective pain was assessed by a ten-point Likert scale, and the anxiety and mood assessment was adapted from the Brief Mood Introspection Scale (BMIS) [8]. Moods assessed by the BMIS were categorized based on positive or negative characteristics. Positive characteristics included: lively, happy, caring, content, gloomy, peppy, calm, loving, and active. Negative characteristics included: sad, tired, gloomy, jittery, drowsy, nervous, and fed up. The BMIS scores were calculated using the University of New Hampshire’s Brief Mood Introspection Scale’s scoring instructions. The four subscores were calculated by visit using the reverse scoring method along the following axes: pleasant-unpleasant, arousal-calm, positive-tired, and negative-relaxed [8]. The BMIS was used to investigate mood and anxiety as it assessed present, transient mood, rather than somatic, autonomic, or chronic symptoms characteristic of the more utilized Hamilton Rating Scale for anxiety.

**Figure 1:**
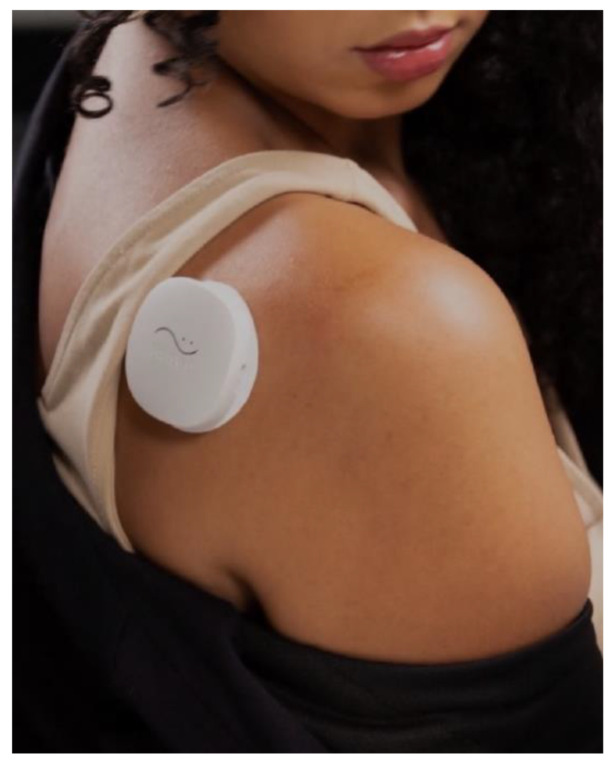
Pulsing heating device, Soovu System.

During the intervention, the focal, pulsing heating devices (The Soovu System, Soovu™) were placed on fourteen locations on the body: a total of four on the back, one on each ventral wrist, one on the dorsal aspect of each hand, one on each proximal shin, one proximal to the lateral ankle on each leg, and one on the dorsal aspect of each foot. The heating device is depicted in Figure 1. Participants underwent three consecutive thirty-minute heating sessions up to 45 °C, interspersed by five-minute cooling periods. The heating devices were connected via Bluetooth with an application made by the manufacturer, and the sessions were manually restarted through the app by the study coordinator. The devices, which have four embedded temperature settings, were initially turned to the highest setting. In the event of participant discomfort, the heating devices were turned down one setting.

Following the heating intervention, the devices were removed, and the participants completed the subjective pain scale assessment and BMIS assessment once again. In addition, each location underwent digital photography, TEWL measurement, and skin colorimeter measurement, allowing for a comparison of skin parameters directly before and after treatment. The participants then attended a follow-up visit 7–10 days later. The follow-up visit consisted solely of digital photography, TEWL measurement, skin colorimeter measurement, and survey completion.

## 3. Results

**Figure 2:**
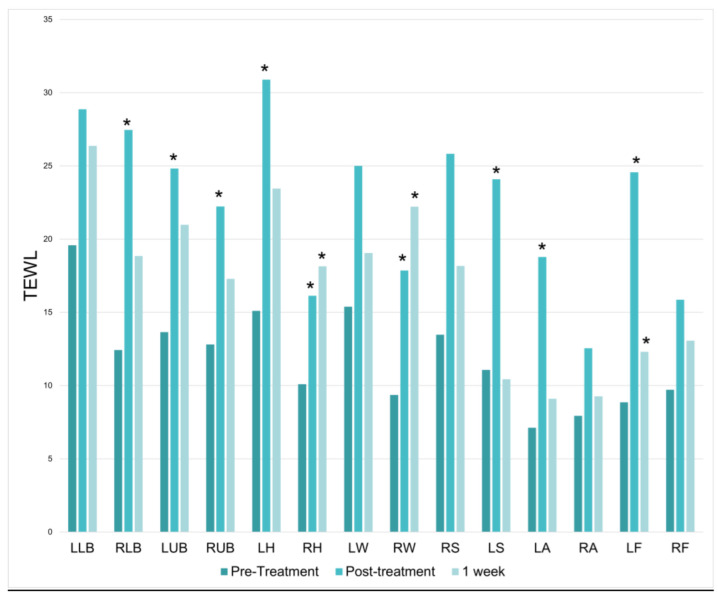
Transepidermal Water Loss (TEWL) baseline, immediately following treatment and one week following treatment. * = *p* < 0.05.

20 women and 2 men enrolled in the study, reflecting skewed gender prevalences on the study interest list. There was a significant increase in TEWL at 9 of the 14 treatment locations immediately following the treatment session; however, significant TEWL increases did not persist at the 1-week mark. On average, there was a 97% increase in TEWL directly after treatment (*p* = 8.04487 × 10^−7^) (Figure 2).

**Figure 3:**
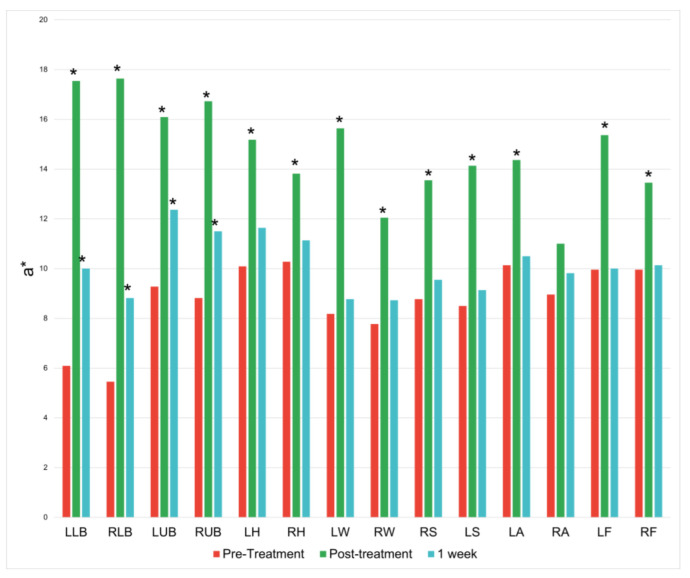
Changes along the red/green coordinate (a*) at baseline, immediately following treatment, and one week following treatment. * = *p* < 0.05.

There was an increase along the red/green axis at 13 of the 14 treatment locations immediately following the treatment session. These increases lessened at the 1-week mark and only persisted significantly at the four treatment locations on the back (Figure 3).

**Figure 4:**
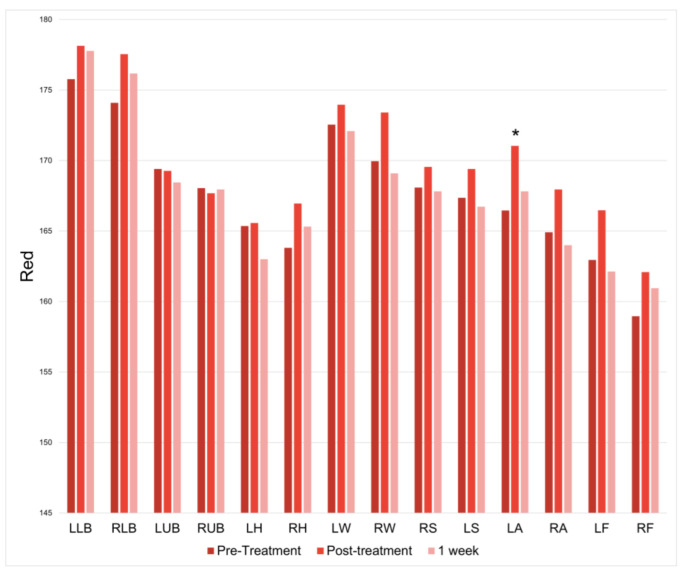
Red at baseline, immediately following treatment, and one week following treatment. * = *p* < 0.05.

There was only a significant increase in redness at the left ankle immediately following treatment (*p* = 0.001126065); however, this increase was not observed at one week post-treatment (Figure 4).

**Figure 5:**
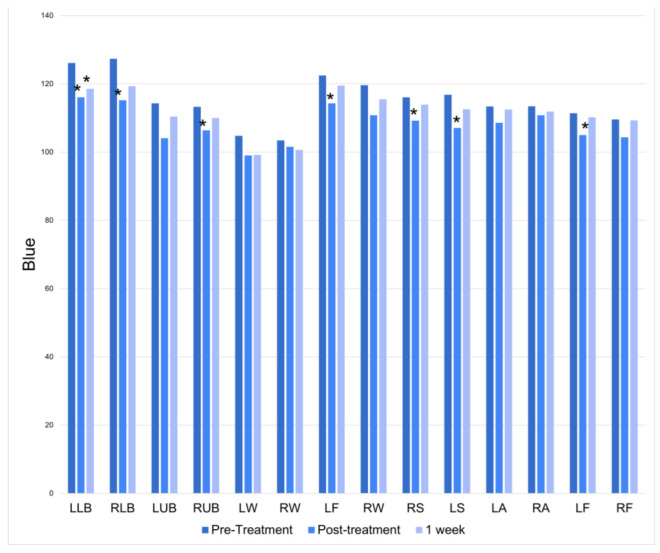
Blue at baseline, immediately following treatment, and one week following treatment. * = *p* < 0.05.

There was a significant increase in blue at half of the treatment locations immediately following the treatment session. At the 1-week follow-up, only the left lower back had a persistently significant increase in blue (Figure 5).

**Figure 6:**
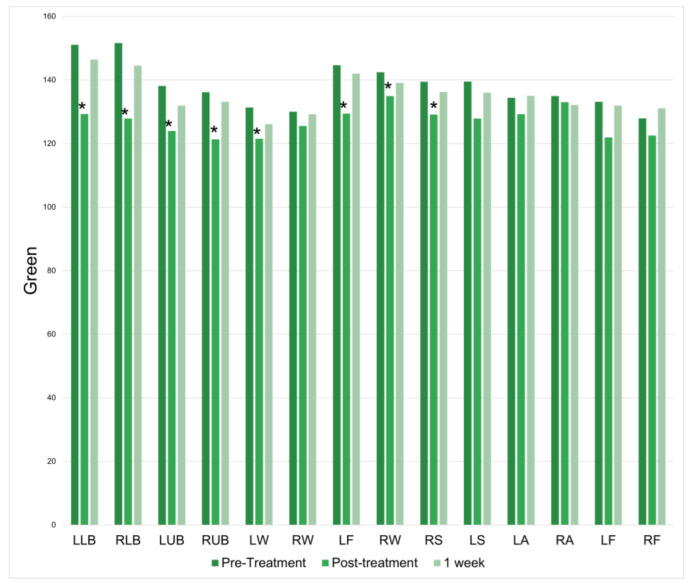
Green at baseline, immediately following treatment, and one week following treatment. * = *p* < 0.05.

There was a significant increase in green at 11 of the 14 treatment locations immediately following the treatment session. However, this change was not found at the 1-week follow-up (Figure 6). 

**Figure 7:**
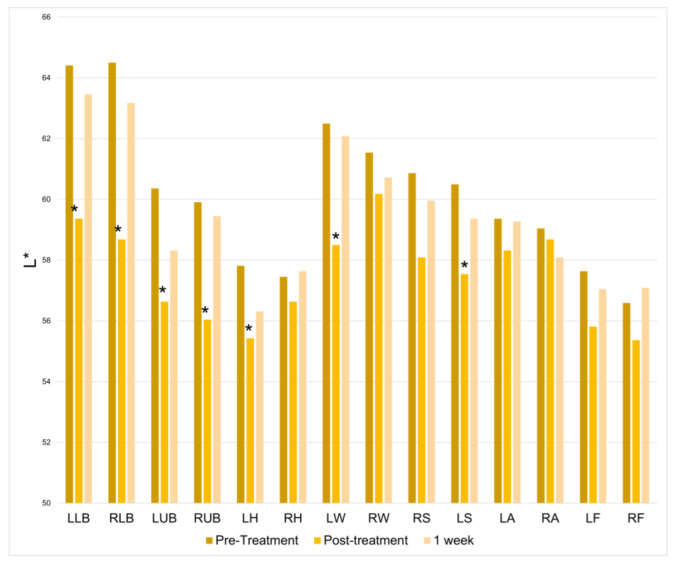
Lightness from black to white (L*) at baseline, immediately following treatment, and one week following treatment. * = *p* < 0.05.

There was a significant increase in lightness at half of the treatment locations immediately following the treatment session. However, this change was not found at the 1-week follow-up (Figure 7). Table 1 summarizes the number of treatment locations depicting significantly increased skin parameter values post-treatment and at one week: TEWL, red/green axis, red, blue, green, lightness.

The heating device was turned down to the second highest setting in 63.6% of the treatments. The most common areas requested for heating to be turned off were the upper and lower back.

**Figure 8:**
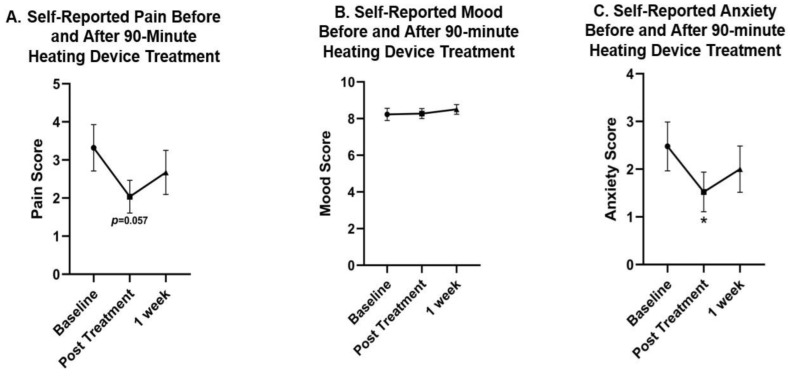
The effect of the pulsed heating intervention on self-reported pain, self-reported mood, and self-reported anxiety at baseline, immediately following treatment, and one week following treatment. (**A**) Self-reported pain: a non-significant 1.29-point decrease in self-reported pain was observed immediately following treatment (*p* = 0.057). Subjective pain remained non-significantly decreased one week following treatment. (**B**) Self-reported mood: no significant differences were observed immediately following treatment or one week following treatment compared to baseline. (**C**) Self-reported anxiety: a statistically significant 38.6% decrease in subjective anxiety was observed immediately following intervention (*p* = 0.0139). Self-reported anxiety remained non-significantly reduced one week following treatment (*p* = 0.1397). * = *p* < 0.05.

There was a 1.29-point decrease in subjective pain immediately following treatment (*p* = 0.057) (Figure 8A). No significant changes were observed in positive or negative mood characteristics. Similarly, there was no significant difference in overall subjective mood between pre- and post-treatment measurements (Figure 8B). However, there was a statistically significant 38.6% decrease in subjective anxiety immediately following the heating device treatment (*p* = 0.0139) (Figure 8C). Although self-reported anxiety remained 19.3% reduced at the one-week follow-up visit, this reduction was not statistically significant compared to baseline measures (*p* = 0.1397).

One subject reported mild blistering three days following treatment but included sites that were unrelated to device placement, and she was deemed to have skin blistering that was not related to the devices. This participant returned for a short, two-cycle heating treatment in 14 adjacent locations to the original treatment locations. The patient tolerated this shorter session without any blistering. Otherwise, there were no other adverse effects reported.

## 4. Discussion

Significant changes in TEWL, the red-green axis, lightness, green, and blue were observed temporarily after treatment in many of the treatment locations, but these effects were typically not observed at the one-week mark. The heating device elicits short-term changes to the skin, but the lack of significant changes to the skin at the one-week mark suggest that the heating devices can be safely used on the skin without any long-term changes in color and water-loss.

This study established the safety of using digital pulsed heating devices when used directly on the skin for three heating cycles. It is important that users of the heating device adjust the heating intensity to the highest level tolerable to prevent temporary discomfort, blistering, or unintended effects on the skin. Shorter heating treatment cycles or lower heat intensity should be considered in patients with thinning skin due to advanced age or underlying skin conditions.

Overall, the results depict a significant reduction in self-reported anxiety immediately, a non-significant reduction in self-reported pain, and no significant changes in self-reported mood immediately following treatment. The decrease in self-reported anxiety following pulsing heating device intervention suggests that heating device therapy may be an effective tool for improving feelings of anxiety. These results were not significantly sustained one week following intervention, suggesting more frequent use may be necessary for maintenance of anxiety improvement. Future studies are required to determine the frequency at which anxiety reduction is sustained.

Typical approaches to managing anxiety involve the use of pharmacological agents, psychological therapies, mindfulness approaches, and exercise [9,10]. Our work expands the options to include device-based heating as another adjuvant to include as potential interventions.

It is interesting to note that the baseline anxiety measures amongst the participants were relatively low and we were able to ascertain a reduction in anxiety scores despite this. Therefore, it will be interesting and warranted to assess the use of digital pulsed heating devices among those that report higher anxiety scores at baseline in future studies.

This study had several limitations. This study served as a pilot study with a smaller sample size, but each person served as their own control, and this increases the power of the study to assess changes over time. This study was designed as an open-label study; it is possible some participants were biased by anticipated or expected outcomes when completing survey materials. Furthermore, participants’ baseline psychiatric medication use was not evaluated, which may impact the anxiety response. Future studies are warranted based on the results discovered here and may include a comparator group such as acupuncture or acupressure to better assess the findings here.

## 5. Conclusions

This prospective, open-label study evaluated the effects of three consecutive thirty-minute treatment cycles with localized heating devices on transepidermal water loss, skin color, subjective pain, mood, and anxiety. Although changes related to water loss and skin color were observed after three treatment cycles, these changes did not persist one week following treatment and therefore suggest safety with up to three consecutive treatment cycles. Because there is increased water loss and changes to skin color immediately following the heating treatment, the exact duration of how long the changes to the skin last should be further studied to improve safety guidelines surrounding the device.

A significant reduction in self-reported anxiety immediately following Soovu heating device intervention was observed, suggesting that heating device therapy may be an effective tool for anxiety improvement. However, maintenance may be required for sustained results. Future research may seek to compare heating device treatment to other anxiety-reducing interventions to assess the device’s clinical utility for anxiety reduction. Overall, the heating device appears to be safe for up to three consecutive heating cycles that were tested.

## Figures and Tables

**Table 1:** Summary of the number of treatment locations with significantly increased skin parameter values post-treatment and at one week.

Parameter	Locations with Significantly Increased Parameter Value Post-Treatment	Locations with Significantly Increased Parameter Value at One Week
TEWL	9/14	2/14
Red/Green axis	13/14	4/14
Red	1/14	0/14
Blue	7/14	1/14
Green	11/14	0/14
Lightness	7/14	0/14

## Data Availability

The data is not available publicy.

## Abbreviations

BMIS	Brief Mood Introspection Scale
NSAIDs	Nonsteroidal anti-inflammatory drugs
TEWL	Transepidermal water loss
